# P-913. Factors Associated with Prolonged Treatment of Uncomplicated Gram-Negative Bacteremia

**DOI:** 10.1093/ofid/ofaf695.1119

**Published:** 2026-01-11

**Authors:** Thomas Hyson, Christina Maguire, Jesal Shah, Amanda Binkley

**Affiliations:** Penn Presbyterian Medical Center, Philadelphia, PA; VCU Health System, Richmond, Virginia; University of Pennsylvania, Philadelphia, Pennsylvania; Penn Presbyterian Medical Center, Philadelphia, PA

## Abstract

**Background:**

Data has shown that seven days of antibiotic therapy is noninferior to 14 days for the treatment of uncomplicated gram-negative rod (GNR) bacteremia and helps combat rising resistance rates. This study evaluated current practice and factors associated with prolonged treatment duration decisions.

**Figure:**
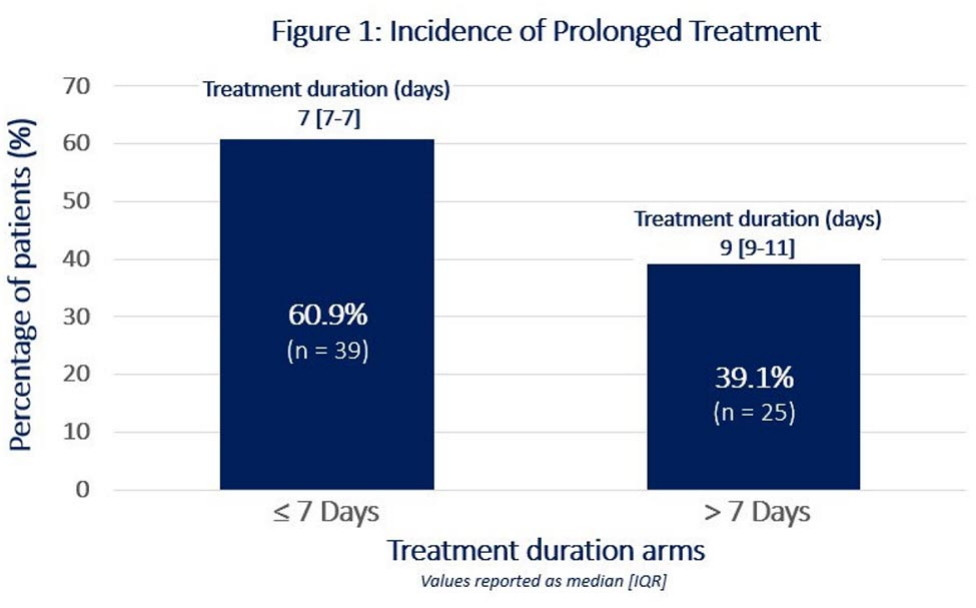

**Figure:**
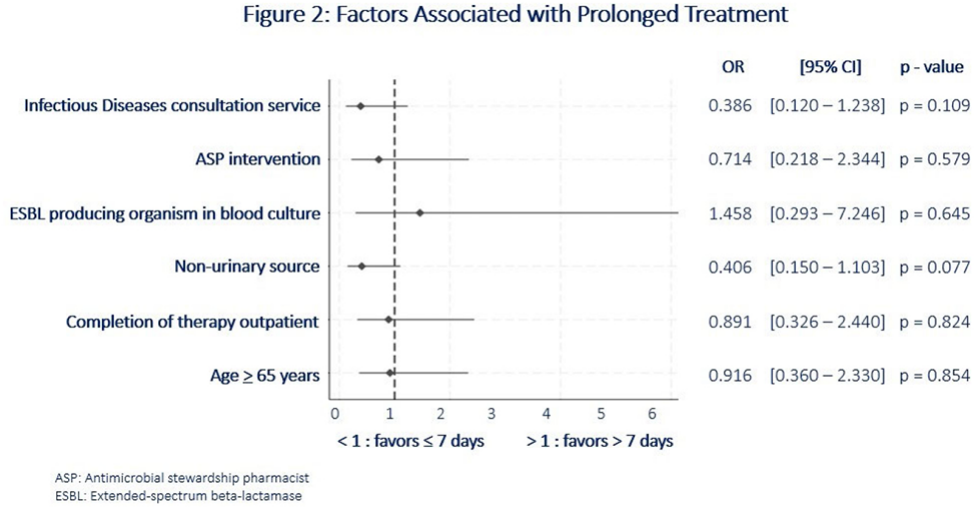

**Methods:**

This retrospective cohort study evaluated adult patients admitted to Penn Presbyterian Medical Center from August 1, 2023, to October 1, 2024. Patients were identified for screening using ILÚM Insight® reports for GNR bacteremia. Patients were included if they met criteria for uncomplicated bacteremia defined by hemodynamic stability, resolution of leukocytosis/leukopenia, and resolution of fever/hypothermia by day five and beyond of appropriate antibiotic therapy based on susceptibility data. The primary outcome of this study was to determine the incidence of treatment durations longer than seven days, while the secondary outcome evaluated factors hypothesized to impact treatment duration decisions.

**Results:**

Of the 280 patients screened, 64 met the inclusion criteria. The cohort consisted of primarily Black males with a median age of 70 years. *Escherichia coli* (43.8%) was the most common bacteria isolated, with lower urinary tract being the most common source of bacteremia (60.9%). Antibiotic treatment durations beyond seven days occurred in 39.1% of patients, with a median duration of nine days (Figure 1). Although nonsignificant, trends toward seven-day treatment courses were seen with non-urinary sources (OR 0.406 [95% CI 0.150 –1.103]; p = 0.077) and Infectious Diseases consultation services (OR 0.386 [95% CI 0.120 – 1.238]; p = 0.109). Cultures with ESBL producing isolates appeared to favor treatment courses beyond seven days (OR 1.458 [95% CI 0.293 – 7.246]; p = 0.645) (Figure 2).

**Conclusion:**

This study demonstrated that most patients at our institution received shorter antibiotic courses for uncomplicated GNR bacteremia, consistent with current supporting literature. Blood cultures with ESBL producing isolates appeared to favor prolonged treatment durations. Although unanticipated, bacteremia from a non-urinary source favored shorter treatment courses. These findings warrant further evaluation and present an area for optimization at our institution.

**Disclosures:**

Amanda Binkley, PharmD, BCIDP, AAHIVP, Shionogi: Advisor/Consultant

